# Trauma Therapists’ Clinical Applications, Training, and Personal Practice of Mindfulness and Meditation

**DOI:** 10.1007/s12671-016-0497-9

**Published:** 2016-02-22

**Authors:** Lynn C. Waelde, Jason M. Thompson, Alicia Robinson, Sierra Iwanicki

**Affiliations:** Palo Alto University, 1791 Arastradero Road, Palo Alto, CA 94304 USA; Eastern Michigan University, Ypsilanti, MI USA

**Keywords:** Mindfulness, Meditation, Trauma therapy, Therapist survey, Therapist training, Psychotherapy

## Abstract

Mindfulness and meditation (MM) are increasingly used in trauma treatment, yet there is little research about therapist qualifications and clinical applications of these practices. We surveyed trauma therapists (*N* = 116) about their clinical uses, training, and personal practice of MM. Most respondents reported use of MM in trauma therapy, primarily MM-related imagery and breathing exercises and mindfulness in session or daily life. Almost a third used mindfulness-based stress reduction, mindfulness-based cognitive therapy, or mindfulness-based relapse prevention. Across all respondents, 66 % were trained by a mental health (MH) professional, 16 % were trained exclusively by a spiritual teacher, and 18 % received no training. On average, therapists used four types of MM. Less than half maintained a personal meditation practice and only 9 % reported practicing daily meditation. Therapists who were trained by a MH professional were more likely to integrate MM into trauma psychotherapy; those who were trained by a spiritual teacher were more likely to teach clients to use MM between sessions and reported more personal practice of MM. Results indicate divergence from standard recommendations for therapist personal practice and professional training in manualized uses; however, there is little guidance about requisite training and personal practice to support individualized uses of MM such as breathing exercises and imagery. Further research should address relationships of therapist training and personal practice to clinical outcomes in MM-informed trauma therapy.

## Introduction

Although clinical trials of mindfulness and meditation (MM) for posttraumatic stress disorder (PTSD) are in the beginning stages, MM-informed interventions are now widely adopted in clinical settings that serve trauma-exposed individuals. A survey of North American psychotherapists found that mindfulness was the third most frequently endorsed theoretical orientation and more than a fifth taught mindfulness-based skills to half or more of their clients (Cook et al. 2010). A survey of Veterans Affairs hospitals found that 72 % offer MM services (VA Office of Research and Development, 2011). Despite this increasing interest, existing research has not addressed implementations of MM among practicing clinicians, in terms of the sources of therapist training and uses in trauma treatment. Given that empirical support for MM in trauma treatment is still emerging and equivocal (e.g., Kearney et al. 2013a; Niles et al., 2012) and there are clinical caveats about the suitability of these practices for persons with PTSD (Vujanovic et al. 2011), understanding current implementations of these practices may guide future research and formulation of treatment and training guidelines.

In clinical settings, MM refers to a spectrum of practices that include techniques promoting mindfulness in everyday life (including mindful awareness of daily activities), sitting mindfulness meditation, and other meditation techniques, such as mantra, compassion, and visualization (Ospina et al., 2007). Most scientific definitions of mindfulness emphasize the observation of present-moment experience (Bergomi et al. 2013). Meditation has been defined as a family of self-regulation practices that focus on training attention and awareness to bring mental processes under greater voluntary control (Walsh & Shapiro, 2006). There is much overlap among different types of meditation; for example, focused attention on a single object, such as the breath, is common to many (Chiesa, 2013) and hence diverse forms of meditation may enhance the capacity to maintain present moment attention. Clinical interventions use MM in diverse forms: Some incorporate techniques designed to cultivate present-moment mindfulness in everyday life and do not use formal sitting meditation as a core treatment component, such as acceptance and commitment therapy (ACT; Hayes & Strosahl, 2010) and dialectical behavior therapy (DBT; Linehan, 1993). Mindfulness-based interventions (MBIs) are a family of psychotherapeutic interventions in which both mindfulness techniques and meditation are conceptualized as core treatment components. MBIs include mindfulness-based stress reduction (MBSR; Kabat-Zinn, 2005) and interventions that combine mindfulness with conventional psychotherapy, such as mindfulness-based cognitive therapy (MBCT; Segal et al. 2002), and mindfulness-based relapse prevention (MBRP; Bowen et al., 2009). It is not clear how these combinations of mindfulness with psychotherapy differ from standard cognitive-behavioral therapies (CBTs), as clinical trials have not provided robust evidence for the comparative effectiveness of MBSR or MBCT versus CBT (Vøllestad et al. 2012; Williams et al., 2014). Other MBIs combine mindfulness with additional components, such as mindful self-compassion (Germer & Neff, 2013) and inner resources for stress, which includes mindfulness, meditation, and breath-focused mantra (Waelde et al., 2008). Outside of the mindfulness domain are meditation programs that emphasize mantra repetition, such as transcendental meditation (TM; Forem, 2012), relaxation response (RR; Benson & Klipper, 2000), and mantram (Lang et al., 2012). Interestingly, in a randomized controlled trial (RCT), TM training was associated with increases in trait mindfulness, which suggests that mindfulness may be a common outcome of multiple types of meditation (Tanner et al., 2009).

Although MBIs have widespread clinical applications, research has only recently begun to address the effectiveness of these practices in trauma therapy. Because mindfulness training is associated with enhanced emotion regulation and non-judgmental acceptance of present moment experience, it may decrease avoidance and reduce physiological hyperarousal (Bormann et al. 2013; Lang et al., 2012), facilitate indirect cognitive-affective exposure (Vujanovic et al., 2011), and reduce trauma-related dissociation by promoting continuity of present-moment awareness (Waelde, 2015). However, it is not known whether MM practices are compatible with standard cognitive-behavioral treatments for PTSD that involve noticing and changing problematic thoughts, rather than non-judgmental acceptance of them (Vujanovic et al., 2011). Investigations of MM in trauma treatment for veterans include MBSR (Bhatnagar et al., 2013; Kearney et al. 2013b; Niles et al., 2012), MBCT (King et al., 2013), loving-kindness meditation (Kearney et al. 2013a), mantram (Bormann et al., 2013), and TM (Brooks & Scarano, 1985; Rosenthal et al. 2011). MBSR has also been investigated with child abuse survivors (Kimbrough et al. 2010). Inner resources for stress was tested for PTSD symptoms with disaster survivors (Waelde et al., 2008) and integrated with psychological first aid and a post-disaster resilience intervention (Hechanova et al. 2015a, 2015b).

Previous investigations of MM for trauma disorders have primarily addressed the effects of manualized MBIs used as stand-alone interventions, rather than evaluating these interventions as adjunctive treatments offered concurrently with standard trauma therapy or individualized uses of MM integrated with standard therapy. However, there are indications that common implementations of MM involve their use as adjuncts in settings where standard psychotherapy is used concurrently (VA Office of Research and Development, 2011). In addition, there are growing indications that MM, rather than being used in manualized formats, is commonly integrated in individualized ways with standard psychotherapies, for example, through the use of mindfulness skills during the course of established PTSD or other psychotherapies (Cook et al., 2010; Vujanovic et al., 2011). There is little guidance about the comparative effectiveness of these three modalities—stand-alone, adjunctive, or integrated practice—or their respective therapist training requirements.

One set of training standards for MBSR and MBCT teachers includes an ongoing daily personal mindfulness practice and at least 12 months of in-depth, rigorous mindfulness teacher training and supervision, which may be provided by a combination of mindfulness teachers and professional clinical trainers (Crane et al., 2012; UK Network of Mindfulness-Based Teacher Trainers, 2010). However, these standards refer to manualized interventions, and there is little specific guidance about trauma therapist qualifications for MM practice, particularly for individualized uses such as the incorporation of breathing exercises or breath-guided imagery into standard therapy. Previous work has investigated both professional and spiritual training sources. In one study, professional mindfulness training enhanced therapists’ mindfulness knowledge and skills, confidence integrating mindfulness into clinical work, and self-ratings of in-session therapist mindfulness (Aggs & Bambling, 2010). An RCT found better pre/postsymptom reductions among patients whose student therapists had participated in meditations with a Japanese Zen master prior to therapy sessions relative to those in the control group (Grepmair et al., 2007). A meta-analysis of MBIs concluded that therapists’ mindfulness experience, but not general clinical training, moderated clinical outcomes (Khoury et al., 2013). As Khoury and colleagues pointed out, a few studies report on therapist MM experience, despite indications that it may be a strong predictor of clinical outcomes, so clearly there is a need to better understand sources of therapist training and personal MM practice.

The present study was designed to examine sources of MM training and degree of ongoing personal practice among practicing trauma therapists. Given the range of MM interventions and techniques that have been investigated for PTSD, we also examined the degree to which therapists incorporate different types of MM interventions, such as standardized interventions (e.g., MBSR and MBCT), religious/spiritual forms (e.g., Zen and kundalini), and secularized techniques (mindful breathing) into trauma treatment. We also examined whether the source of MM training (none, professional, or spiritual) was associated with (1) clinicians’ use of standardized MM interventions; (2) clinical applications of MM such as use as a stand-alone treatment, use as an adjunctive treatment or integration into standard trauma therapy; and (3) frequency of therapists’ personal MM practice among therapists who used MM in trauma therapy.

## Method

### Participants

Data for this study were drawn from a larger survey about secondary traumatization and self-care strategies of practicing trauma therapists distributed to the electronic mailing lists of the Association for Addiction Professionals (NAADAC), American Psychological Association (APA), and International Society for Traumatic Stress Studies (ISTSS) in 2011. The survey was sent to the NAADAC membership; APA Division 12 Society of Clinical Psychology Sections 2: Clinical Geropsychology and 10: Graduate Student and Early Psychologists; and special interest groups of ISTSS: trauma assessment and diagnosis, research methodology, graduate student/postdoctoral fellows, and trauma and substance use disorders (Iwanicki et al. 2014). Of the 208 responses to this survey, 63 were excluded because they were blank, yielding a response rate of 70 %, and 29 were excluded for missing data (21 respondents indicated that they did not use MM clinically and skipped the rest of the MM questions, 2 reported some clinical use of MM but omitted the other MM questions, and 6 did not answer any MM questions). Participants were the remaining *N* = 116 respondents.

Respondents came from all three recruitment sources: NAADAC (*n* = 70; 60.3 %), APA (*n* = 33; 28.4 %), and ISTSS (*n* = 13; 11.2 %). Most participants were women (*n* = 76; 65.5 %) and Caucasian-Americans (*n* = 102; 87.9 %). Other ethnic groups included Asian-Americans (*n* = 3; 2.6 %), Latino-Americans (*n* = 3; 2.6 %), African-American (*n* = 1; 0.9 %), and those of mixed ethnicity (*n* = 7; 6.0 %). Most participants were married (*n* = 71; 61.2 %) or single (*n* = 13; 11.2 %); the rest identified as divorced or widowed (*n* = 11; 9.5 %), in a relationship with an opposite-sex partner (*n* = 17; 14.7 %) or in a relationship with a same-sex partner (*n* = 4; 3.5 %). Half of the sample identified themselves as licensed professional counselors (*n* = 58; 50.0 %); the rest were psychologists (*n* = 26; 22.4 %), social workers (*n* = 14; 12.0 %), students (*n* = 9; 7.8 %), addictions counselors (*n* = 8; 6.9 %), and a psychiatrist (*n* = 1; 0.9 %). All participants averaged 21.9 h (*SD* = 14.9) a week of direct client contact; they had been engaged in clinical work with traumatized clients for an average of 14.0 years (*SD* = 10.3, range = <1–40 years). On average, significant proportions of their caseloads included clients who had been sexually, physically, or emotionally abused or neglected (*M* = 33.3, *SD* = 28.2; *M* = 37.2, *SD* = 29.1; *M* = 50.38, *SD* = 30.4; *M* = 32.3, *SD* = 27.5, respectively).

### Procedure

An invitation to participate in an online survey of self-care strategies was emailed to members of three professional organizations, NAADAC, APA, and ISTSS. The survey was posted online on a secure website and participants followed a link in the invitation email to the survey web address. Respondents were not compensated for their participation. The Human Subjects Review Committee of Eastern Michigan University approved the research protocol.

### Measures

A demographics and clinical practice questionnaire included questions about gender, relationship status, ethnicity, profession, number of hours per week of client contact, years of experience as a trauma therapist, and proportion of caseload involving traumatized clients. Three multiple-answer format items assessed sources of MM training, uses of MM in trauma treatment, and types of MM techniques used in trauma therapy, and included “none” and “other” as response options (see response option stems in Table 1). The item assessing sources of MM training asked, “Where did you get training in the use of mindfulness/meditation (MM) with psychotherapy clients? (Check all that apply).” Responses options were, “I have not received any formal training in MM,” “I apply what I learned about MM from a spiritual teacher,” “Training for mental health professionals provided by a spiritual teacher,” and “Training for mental health professionals provided by a metal health professional.” The item assessing uses of MM in trauma therapy was, “I incorporate MM practice with trauma therapy clients in the following ways (check all that apply).” Eight response options included, “I do not use or refer clients to MM instruction in my trauma therapy practice,” and “I integrate MM into standard trauma psychotherapy sessions.” The item assessing types of MM techniques used in trauma therapy was, “In my trauma therapy practice, I teach MM techniques drawn from the following sources (check all that apply).” Seventeen response options allowed respondents to indicate which of six standardized MM interventions (e.g., MBSR, MBCT, and RR), five religious forms of meditation (e.g., Zen Buddhist and kundalini), and six MM techniques (e.g., mantra meditation) they used. Respondents also indicated how frequently they personally practiced MM, choosing one of five response options ranging from 0 (I do not have a personal mindfulness/meditation practice) to 4 (I have a daily sitting meditation practice).

**Table 1:** Analyses of uses of MM and frequency of therapists’ personal practice by source of therapists’ MM training (*N* = 116)

	None	Spiritual teacher	MH professional	χ^2^	*p*	Φ
	*n* = 21	*n* = 18	*n* = 77^a^			
Uses of MM in trauma therapy^b^						
No MM use or referrals	5 (23.8)	0 (0)	2 (2.6)	11.27	.004	.35
Refer to MM classes in community	0 (0)	4 (22.2)	16 (20.8)	8.88	.012	.22
Adjunct to standard trauma treatment	4 (19.0)	4 (22.2)	27 (35.1)	2.78	*ns*	.15
Integrate into trauma treatment	8 (38.1)	5 (27.8)	51 (66.2)	11.91	.003	.32
Teach mindfulness for use in daily life	12 (57.1)	16 (88.9)	50 (64.9)	5.72	.057	.21
Teach sitting MM practice	6 (28.6)	11 (61.1)	30 (39.0)	4.47	*ns*	.20
Provide recordings of guided practice	7 (33.3)	4 (22.2)	26 (33.8)	.97	*ns*	.09
Stand-alone treatment	1 (4.8)	0 (0)	3 (3.9)	1.40	*ns*	.08
	*n* = 16	*n* = 18	*n* = 74	F	*p*	η^2^
Frequency of personal MM practice^c^				9.41	< .001	.15
No personal practice	5 (31.3)	0 (0)	9 (12.2)			
Mindfulness practice in daily life only	9 (56.3)	6 (33.3)	25 (33.8)			
Sitting meditation a few times a month	0 (0)	2 (11.1)	23 (31.1)			
Sitting meditation a few times a week	2 (12.5)	4 (22.2)	13 (17.6)			
Daily sitting meditation practice	0 (0)	6 (33.3)	4 (5.4)			

*MM* mindfulness and/or meditation, *MH* mental health

Statistics are *n*s and percentages for each MM training source group unless noted otherwise

^a^Personal MM practice data were missing for *n* = 1 in the MH professional group

^b^Participants could select more than one answer for uses of MM, so statistics are shown for those endorsing each usage

^c^Participants who do not use MM in trauma therapy (*n* = 7) were excluded from this analysis

### Data Analyses

Likelihood ratio chi-square analyses were used to compare the relationship of training source to (1) use (vs. non-use) of standardized MBIs and (2) use (vs. non-use) of particular strategies of MM intervention. A univariate ANOVA with post hoc Bonferroni tests examined the relationship of training source to frequency of personal practice among those who reported using MM in trauma treatment. We used Pearson correlations to evaluate age, gender, and years of experience as a trauma therapist for inclusion as covariates in the ANOVA but none were significantly correlated with the dependent variable (frequency of MM practice) and were not included.

## Results

Among the 145 respondents with non-blank surveys, 117 (80.7 %) indicated that they used MM in some form in trauma therapy. Of the *N* = 116 respondents with complete data, *n* = 108 (93.9 %) were using MM in some form in their trauma therapy practice.

Most participants taught mindfulness techniques for the client to use during daily life (*n* = 78; 67.2 %) and integrated MM into standard psychotherapy sessions (*n* = 64; 55.2 %). A minority taught sitting mindfulness or other sitting meditation practice for clients to use between sessions (*n* = 47; 40.5 %), but only 31.9 % (*n* = 37) provide guided meditation audio or video recordings for clients to use between sessions. Almost a fifth of the therapists (*n* = 20; 17.2 %) referred clients to MM classes in the community. Some therapists used MM as an adjunct to standard psychotherapy (*n* = 35; 30.2 %), but only 3.4 % (*n =* 4) regarded it as a stand-alone therapy.

Most of this sample of trauma therapists reported receiving MM training from MH professionals (*n* = 77; 66.4 %); of these 77 participants, 17 had also received instruction from a spiritual teacher. Of the 15.5 % (*n* = 18) who reported receiving training exclusively from spiritual teachers, 13 reported applying what they had learned in a spiritual context to clinical work and 5 had received training from a spiritual teacher that was offered specifically for MH professionals. A minority of the sample reported no training in MM (*n* = 21; 18.1 %).

MM-related imagery was the most commonly used technique in trauma therapy, with a majority reporting they used imagery that focuses on the breathing or body (*n* = 71; 61.2 %) or outside the body (e.g., a favorite place) (*n* = 71; 61.2 %). A majority also reported MM-related breathing or *pranayama* exercises (*n* = 69; 59.5 %). Use of non-MBSR mindfulness was reported by 40.5 % (*n* = 47). Each of three standardized MM interventions were practiced by almost a third of the sample: MBCT (*n* = 36; 31 %), MBSR (*n* = 34; 29.3 %), and RR (*n* = 32; 27.6). TM was used by 6.9 % (*n* = 8). A minority of therapists reported incorporating practices drawn from Zen Buddhism (*n* = 16; 13.8 %), Vipassana (*n* = 7; 6 %), mantra repetition (*n* = 10; 8.6 %), and Hatha yoga (*n* = 10; 8.6 %). Less than 5 % reported using MM techniques drawn from each of the following practices: ACT, DBT, MBRP, inner resources for stress, somatic re-experiencing, Sahaja yoga, Sudarshan Kriya yoga, Kundalini yoga, Neelakantha meditation, and prayer. Therapists reported using an average of 3.75 types of MM (*SD* = 2.31). Therapists who used MBCT reported using an average of 5.11 (*SD* = 3.01) total types of MM; therapists using the other most common standardized interventions reported these average numbers of MM uses: MBSR (*M* = 5.01, *SD* = 2.99), RR (*M* = 5.13, *SD* = 3.06), and TM (*M* = 6.13, *SD* = 5.33).

Chi-square analyses indicated that source of therapist training (none, spiritual teacher only, or MH professional) was not associated with usage of MBCT, MBSR, RR, or TM. Chi-square analyses using four training groups (none, spiritual teacher only, MH professional, and both spiritual teacher and MH professional) produced very similar results, so the three-group results are presented. Chi-square analyses in Table 1 indicate that MM training was related to greater clinical application of MM: A smaller proportion of those who had received no training used MM clinically and referred clients to MM classes in the community than those trained by a spiritual teacher or by a MH professional. Those trained by a MH professional integrated MM into standard psychotherapy to a greater extent than those without training or with spiritual instruction. However, those with spiritual training encouraged between-session practice, in the forms of mindfulness practice in daily life (a trend at *p* = .057), more than those with no training or training by a MH professional.

Among the *n* = 108 therapists who used some form of MM in trauma therapy, 12.8 % (*n* = 14) had no personal practice and 36.7 % (*n* = 40) reported mindfulness practice in daily life but no sitting practice. Only 9.3 % (*n* = 10) practiced daily sitting meditation. The rest practiced a few times per week (*n* = 19; 17.6 %) or a few times per month (*n* = 25; 23.1 %). As shown in Table 1, ANOVA results indicate that the type of training received was significantly related to the therapists’ degree of personal MM practice. Post hoc analyses showed that those who received no MM training practiced MM less often than those who had been trained by a spiritual teacher (*p* < .001) or a MH professional (*p* = .04). However, those who reported training by a spiritual teacher reported significantly more personal practice time than those who received training from a MH professional (*p* = .01).

## Discussion

The majority of respondents to this online survey of practicing psychotherapists reported using MM in some form in their trauma therapy practice. Almost a third of the sample reported use of standardized MBIs such as MBCT, MBSR, or RR. The high degree of MBI usage is surprising given that the above review indicates that there were few clinical trials of MM for PTSD prior to mid-2011, when this survey was conducted. Much previous research has addressed the gap between research and practice, indicating that practicing clinicians may not consider the evidence base for interventions in clinical decision making (Boisvert & Faust, 2006; Borntrager, Chorpita, Higa-McMillan, & Weisz, 2009). It is possible that the evidence base for MM may be less salient to clinicians than that for other interventions because of its association with Hindu and Buddhist philosophical frameworks that lie outside of mainstream Western psychology (Sedlmeier et al., 2012). MM interventions are a hybrid of Western psychological and Eastern philosophical and religious traditions, and consequently therapists may consider both empirical evidence and spiritual traditions in formulating clinical applications. As an example, MBSR training guidelines require MBSR teachers and trainers to be dharma students (Kabat-Zinn 2016), mandating integration of MBSR clinical applications with Eastern philosophy. Although there has been much scholarship on the meeting of Eastern and Western perspectives, achieving a well-conceived integration in clinical practice may be difficult without development of empirically informed training and treatment guidelines.

The findings about sources of MM training also suggest that clinical MM diverges from conventional psychotherapy in important ways. Although a majority of therapists received MM training from a MH professional, more than a third had sought instruction from a spiritual teacher and a minority had training exclusively from a spiritual teacher. Spiritual teachers may provide needed training for therapists to practice in this specialty area; indeed, a personal MM practice is considered requisite for MBI interventionists. The current study indicated that therapists trained by MH professionals integrated MM into conventional psychotherapy significantly more than therapists trained by spiritual teachers. This finding is unsurprising because therapists trained by MH professionals could reasonably be expected either to have explicitly developed strategies for the integration of MM with conventional psychotherapy or to have conceptualized MM within a paradigm that was more conventionally psychotherapeutic than specifically meditation-related. In addition, spiritual-teacher-trained therapists reported more personal practice and more frequently taught the use of between-session practice than the other two groups (although this latter finding was a trend at *p* = .057). Although we might expect that therapists irrespective of training background would encourage between-session practice consistent with standard recommendations regarding therapy homework, it may be that therapists with a consistent personal practice may be better equipped to teach between-session practice to clients. It is possible that those who received instruction from spiritual teachers have a deeper or more long-term commitment to MM practice. Professional MM training is inherently time-limited, whereas instruction by spiritual teachers may take place over many years. Therapists who received training from both a MH professional and a spiritual teacher reported patterns of MM uses and personal practice that were similar to therapists trained only by MH professionals. However, the current study did not assess the extent of previous training, so future work should examine the relationships of duration and sources of previous MM training, whether spiritual, professional, or both, to MM treatment characteristics. In the current study, endorsements of spiritual training did not specify whether the training was provided in religious (e.g., Buddhist or Hindu) or not explicitly religious contexts (e.g., MBSR). This clarification about training source could inform work about integration of religious versus non-religious approaches to mindfulness into psychotherapy (Grossman & Van Dam, 2011; Purser, 2015; Van Gordon et al. 2015).

The most frequent MM use was teaching mindfulness techniques in everyday life, and only a minority taught sitting meditation practice for clients to use between sessions or provided materials to structure between-session practice. This usage pattern is inconsistent with findings that the amount of sitting meditation practice, but not mindfulness practice in daily life, is associated with symptom improvements as a result of MM training (Carmody & Baer, 2008). Despite the fact that most studies of MBIs for PTSD have involved MM as the primary treatment component, only a few therapists used MM as a stand-alone treatment and about half integrated MM into conventional trauma therapy. These findings reflect a disparity between the evidence base and clinical practice, as existing MBI clinical trials provide little guidance about how to integrate MM into standard therapy. The types of MM practice in use also reflect this disparity. Less than a third of respondents used standard MBIs and the types of MM in use represent a variety of secular and religious approaches. Mindful awareness of breathing, breathing-based imagery, and generic (non-MBSR) mindfulness were among the most common techniques integrated into therapy. Therapists were eclectic, reporting an average of almost four types of MM. Use of standardized MBIs was no barrier to eclecticism because those who reported using any of the four most common MBIs (MBCT, MBSR, RR, or TM) averaged more than five total types of MM practice. Likewise, use of standardized MBIs was unrelated to source of therapist MM training. The diversity of uses and types of MM may reflect therapist preferences for flexibility and modularity in treatment approaches over adherence to treatment manuals (Borntrager et al., 2009). Development of MM for use in trauma therapy should address the need for clinician flexibility.

Examination of the training and personal practice results among those who used MM in trauma therapy also indicates notable divergences from conventional recommendations. Most of the therapists who reported having no MM training reported use of MM in trauma therapy. Those with no training also had significantly less personal practice of MM than those with some training. Although MBI therapist guidelines typically recommend daily sitting MM practice, few therapists reported personal practice of that intensity. Half did no sitting meditation practice at all and 41 % reported only occasional sitting practice, with only 9 % reporting daily sitting meditation. The findings about lack of MM training are of particular concern because legal and ethical standards require psychotherapists to demonstrate competence in the form of education, professional training, supervised experience, and continuing education (Pope & Vasquez, 2011). At present, it is unknown whether training from professional MH sources is adequate to teach competent mindfulness practice and whether training from sources outside of professional contexts is adequate to achieve integration with evidence-based practice. A qualitative study of MBI teacher characteristics found that both therapists and clients emphasized the importance of the therapist’s own ongoing meditation practice and capacity to embody mindfulness principles (van Aalderen et al. 2012), suggesting that therapists without training and a dedicated personal practice may achieve less than optimal therapy outcomes. However, it is still unclear whether and to what extent therapists’ personal MM practice is required for successful therapy, particularly for the use of individualized, non-manualized techniques such as breathing exercises and imagery. There is some suggestion that mindfulness is a common psychotherapeutic factor that is already integrated into diverse psychotherapy approaches (Martin, 1997). If MM techniques such as present-moment awareness are common to diverse psychotherapies, then specialized MM training and personal practice may be unnecessary. However, scholarship has yet to examine whether mindfulness techniques and standard psychotherapy tools, such as those promoting emotional awareness and exposure, are overlapping or identical. Future research should address unique components of mindfulness training and the degree and sources of training needed to produce competent practice and successful treatment outcomes.

The study used a convenience sample of therapists who were members of electronic mailing lists of three professional organizations. It was not possible to calculate an overall response rate because confidentiality requirements precluded a count of the number who received or reviewed the invitation to participate. Although it is a common practice for psychotherapist surveys to use mailing lists of professional organizations (e.g., Boisvert & Faust, 2006; Szkodny et al. 2014) or professional magazines (Cook et al., 2010), probability sampling of the population of practicing psychotherapists (such as all those registered/licensed in a particular area) would permit stronger study generalizations. The current sample, although not demonstrably representative, was predominantly Caucasian-American and female with an average of 14 years of clinical experience and was demographically similar to a North American survey of psychotherapists in which the modal respondent was a Caucasian-American woman with 15 years of experience (Cook et al., 2010). Further, the completion rate of 70 % among those who consented to participate was similar to the 72 % completion rate obtain by Cook and colleagues. A larger, representative sample would permit comparisons of gender, ethnoracial, and professional subgroups to broaden knowledge beyond the modal mid-career Caucasian woman respondent. Half of the sample identified themselves as licensed professional counselors, so the results may not generalize to settings where other types of mental health professionals predominate. In addition, although all participants worked with traumatized clients and reported on applications of MM in trauma therapy, the survey did not include questions about the specific types of trauma therapy being used nor did it clarify the extent to which implementations of standardized MBIs were adherent to their respective treatment manuals. Future surveys should inquire about the types of trauma therapy and MBIs being practiced to shed light on the degree to which MM are being applied in the context of specific types of trauma treatments. Future surveys should also inquire about the nature of therapists’ personal MM practice, including the types of daily life and sitting practice, to illuminate relationships with therapy outcomes.
